# The Prophylactic Effect of Vitamin C and Vitamin B12 against Ultraviolet-C-Induced Hepatotoxicity in Male Rats

**DOI:** 10.3390/molecules28114302

**Published:** 2023-05-24

**Authors:** Azza A. Attia, Huda A. Hamad, M. Adel Fawzy, Samar R. Saleh

**Affiliations:** 1 Zoology Department, Faculty of Science, Alexandria University, Alexandria 21515, Egypt or azzaaattia@hotmail.com (A.A.A.); khirallahhuda@gmail.com (H.A.H.); 2 Zoology Department, Faculty of Science, Omar Al-Mukhtar University, Al Bayda 00218, Libya; 3 Physics Department, Faculty of Science, Alexandria University, Alexandria 21515, Egypt; salem_adelfawzy@yahoo.com; 4 Biochemistry Department, Faculty of Science, Alexandria University, Alexandria 21515, Egypt

**Keywords:** UVC radiation, antioxidants, inflammatory markers, apoptosis, caspase-3, DNA fragmentation, ultrastructural examination

## Abstract

Ultraviolet C (UVC) devices are an effective means of disinfecting surfaces and protecting medical tools against various microbes, including coronavirus. Overexposure to UVC can induce oxidative stress, damage the genetic material, and harm biological systems. This study investigated the prophylactic efficacy of vitamin C and B12 against hepatotoxicity in UVC-intoxicated rats. Rats were irradiated with UVC (725.76, 967.68, and 1048.36 J/cm^2^) for 2 weeks. The rats were pretreated with the aforementioned antioxidants for two months before UVC irradiation. The prophylactic effect of vitamins against UVC hepatotoxicity was evaluated by monitoring the alteration of liver enzyme activities, antioxidant status, apoptotic and inflammatory markers, DNA fragmentation, and histological and ultrastructural alterations. Rats exposed to UVC showed a significant increase in liver enzymes, oxidant–antioxidant balance disruption, and increased hepatic inflammatory markers (TNF-α, IL-1β, iNOS, and IDO-1). Additionally, obvious over-expression of activated caspase-3 protein and DNA fragmentation were detected. Histological and ultrastructural examinations verified the biochemical findings. Co-treatment with vitamins ameliorated the deviated parameters to variable degrees. In conclusion, vitamin C could alleviate UVC-induced hepatotoxicity more than vitamin B12 by diminishing oxidative stress, inflammation, and DNA damage. This study could provide a reference for the clinical practice of vitamin C and B12 as radioprotective for workers in UVC disinfectant areas.

## 1. Introduction

Radiation has become one of the most important challenges in the biological environment and is a concern for human beings [1]. Ultraviolet (UV) radiation is a carcinogen with well-known genotoxic and mutagenic effects [2]. UV radiation is divided into three main categories based on its wavelengths and energy: UVA (315–400 nm), UVB (280–315 nm), and UVC (200–280 nm) [3,4]. UVC, being eco-friendly, is a well-known disinfectant for air, water, and surfaces, which is used to prevent the spread of certain infectious diseases, including coronavirus [5,6]. The UVC wavelengths, particularly 254 nm, are well known for their germicidal ability to damage the genetic material (DNA or RNA) of pathogenic microorganisms [7,8]. Nucleic acids typically absorb wavelengths ranging from 200 to 300 nm. Therefore, UVC rays are strongly and directly absorbed by nucleic acids. UVC absorption into pathogen genomes triggers radiolytic cleavage or radical reactions and dimerization of pyrimidine residues, which consequently affect the correct transcription and replication of RNA and DNA, respectively, and eventually lead to microorganisms’ inactivation [6,8,9,10,11]. During the coronavirus disease 2019 (COVID-19) pandemic, UV rays, particularly UVC, were widely used to disinfect skin areas, clothes, frequently touched surfaces, and other objects in hospitals and food-processing facilities. While UVC rays have been shown to effectively inactivate coronaviruses, UVC technological devices have some limitations [10,11,12]. Excessive exposure to UVC radiation may harm human health, including tissue damage, corneal burns, and snow blindness, in addition to ocular, conjunctival, and skin irritation [3,12,13,14,15]. Therefore, UV rays should be used cautiously and not directly on human skin. Various guidelines recommend that UV rays should not be used as a sole agent for disinfection but as an adjunct to the latest standard disinfecting procedures. Moreover, studies deduced that workers’ safety also needs to be addressed in the near future [8,10]. Recently, Pereira and Braga [11] deduced many environmental and health safety issues concerning the use of UVC irradiation in disinfection. The study reported the potential risks of damaging skin and eyes and even possible carcinogenic effects. Moreover, the study suggested the use of a UVC disinfection system that does not require human intervention or wearing UVC light protection equipment to prevent these risks.

Furthermore, UVC exposure produces several reactive molecules, including superoxide radicals (O_2_^•−^), hydroxyl radicals (·OH), and hydrogen peroxide (H_2_O_2_). These molecules attack important macromolecules, including cellular proteins, nucleic DNA, and membrane lipids, disrupting various physiological cellular responses and ultimately causing cell death. Therefore, UVC can promote the production of reactive oxygen species (ROS), which stimulate oxidative stress and alter the genome integrity [4,13,16].

The body has developed numerous enzymatic and non-enzymatic defense mechanisms against oxidative damage [17,18]. The enzymatic mechanism comprises free radical scavengers, including catalase, superoxide dismutase (SOD), catalase, and glutathione-dependent enzymes such as glutathione- S-transferase (GST), glutathione peroxidase (GPx), and glutathione reductase (GR), which are assumed to be the first line of cellular defense against ROS’ damaging effects [19,20]. The non-enzymatic mechanism involves certain endogenous antioxidants (reduced glutathione (GSH), tocopherol, thioredoxin, etc.), which are found in the body, as well as certain exogenous antioxidants that are ingested by the body (vitamins E, C, flavonoids, etc.) [18,21]. Avti and Pathak [22] reported that endogenous antioxidant levels are markedly elevated by low-dose radiation in different tissues, including the liver, brain, spleen, and testes. Therefore, free-radical scavenging enzymes are used as biomarkers of oxidative stress.

Considering all this, there is a significant need to find a compound, especially with antioxidant and anti-inflammatory properties, that could protect internal organs from UVC radiation. Many natural antioxidant products with interesting strategies for preventing oxidative damage contribute to the pathophysiology and histopathology of many illnesses [17]. Antioxidants affect biological systems in several ways, including metal ion chelation, electron donation, the modulation of gene expression, or functioning as co-antioxidants [23,24].

Vitamins are essential micronutrients that play a crucial role in cellular metabolism, and a lack of them can lead to illness. In addition to their role as nutrients, vitamins are becoming increasingly known to modulate gene expression and signal transduction when consumed at pharmacological concentrations [25]. Vitamin C (ascorbate) is one of the most powerful antioxidants. Vitamin C is utilized to cure several illnesses and prevent oxidative-stress-induced DNA mutation [17,26,27,28]. Because of its excellent water solubility, vitamin C has a unique ability to “scavenge” aqueous peroxy radicals that destroy the membrane lipids [18,29,30,31]. Moreover, vitamin C can function both intracellularly and extracellularly and reduce GSH, which acts through different mechanisms to safeguard it [32]. Vitamin C’s hepatoprotective properties are well-documented through different mechanisms. Vitamin C was reported to normalize the levels of serum aspartate aminotransferase (AST), alanine aminotransferase (ALT), and malondialdehyde (MDA) in intoxicated animals. It enhances the free radical scavenging activities of SOD, catalase, and GPx, limiting lipid peroxidation, fibrosis, cirrhosis, and hepatic inflammation [33,34,35,36].

Vitamin B12, or cobalamin, is a water-soluble and vital micronutrient essential for cell homeostasis, hematopoiesis, and immunity. Vitamin B12 is involved in cell growth, myelin, DNA, and erythrocyte synthesis [37,38]. Cobalamins have been shown to regulate inflammatory cytokines and act as antioxidants [39,40,41,42,43,44]. Vitamin B12 directly scavenges ROS, particularly superoxide, and indirectly stimulates ROS scavenging by maintaining spare GSH and enhancing cytosolic antioxidant bioavailability [41,45]. Moreover, Vitamin B12 controls the synthesis of anti-inflammatory cytokines and growth factors, reducing systemic inflammation [46].

The liver is a critical organ in the human body that regulates the synthesis of almost every plasma protein, including, among others, albumin, binding globulins, protein C, protein S, and intrinsic and extrinsic coagulation factors [47]. The liver also performs various essential functions, including the metabolism of carbohydrates, the storage of vitamins, the synthesis of bile and albumin, and the secretion of several hormones. Additionally, it is essential for immunological function, blood filtration, and detoxification [48].

The present study aimed to determine the potential prophylactic role of antioxidant vitamins C and B12 against hepatotoxicity in male rats exposed to UVC by detecting serum liver enzymes and hepatic oxidative stress markers. Furthermore, the study examined, for the first time, the impact of vitamins C and B12 on the intracytoplasmic production of proinflammatory cytokines in the liver tissues of irradiated rats. Additionally, the study was realized by the histological and ultrastructural examination of the liver cells.

## 2. Results

### 2.1. The Effects of UVC Irradiation, Vitamin C, and Vitamin B12 on Serum Parameters

**Figure 1:**
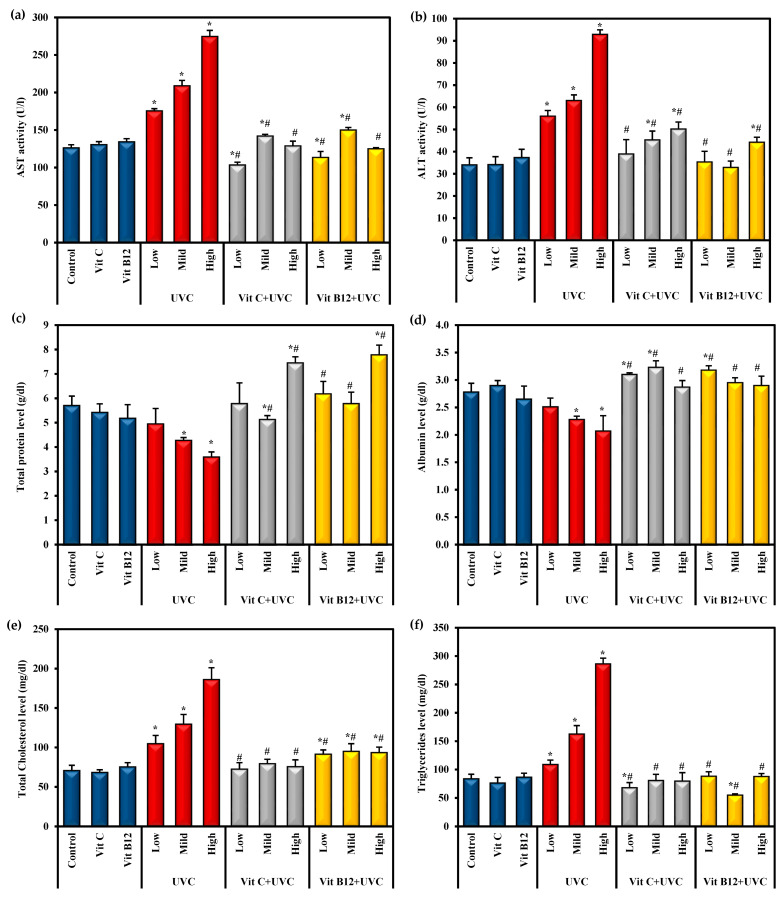
The effects of UVC irradiation, vitamin C, and vitamin B12 on serum parameters. (**a**) ALT activity, (**b**) AST activity, (**c**) total protein level, (**d**) albumin level, (**e**) total cholesterol level and (**f**) triglycerides level. Values are presented as means ± SD of ten rats. One-way analysis of variance (ANOVA) was utilized, followed by a post hoc test (Tukey) for pairwise comparisons. *: Statistically significant compared to the control group at *p* ≤ 0.05. #: Statistically significant compared to the corresponding UVC group at *p* ≤ 0.05.

Rats exposed to UVC irradiation showed a marked hepatotoxic effect, as indicated by a significant (*p* ≤ 0.05) increase in ALT and AST activities and total cholesterol and triglyceride levels associated with a significant (*p* ≤ 0.05) decline in total protein and albumin levels compared to control rats (Figure 1). Low-dose UVC- intoxicated rats showed a non-significant change in total protein and albumin levels compared to the control values. In contrast, rats exposed to the high dose of UVC revealed a significant alteration in these parameters compared to low and mild UVC doses (Figure 1).

The administration of vitamin C or B12 for eight weeks before UVC irradiation resulted in a significant (*p* ≤ 0.05) prophylactic effect in the liver function parameters compared to UVC-irradiated rats. It was found to overcome the hepatotoxic effect of UVC light exposure. Moreover, the administration of vitamin C and vitamin B12 before exposure to different doses of UVC was found to recover the levels of most of those parameters to normal values, approaching those of the control groups. Administration of either vitamin C or B12 to normal rats showed a non-significant change in these parameters (Figure 1).

### 2.2. The Effect of UVC Irradiation, Vitamin C, and Vitamin B12 on the Oxidative Stress Parameters in Liver Tissue

**Figure 2:**
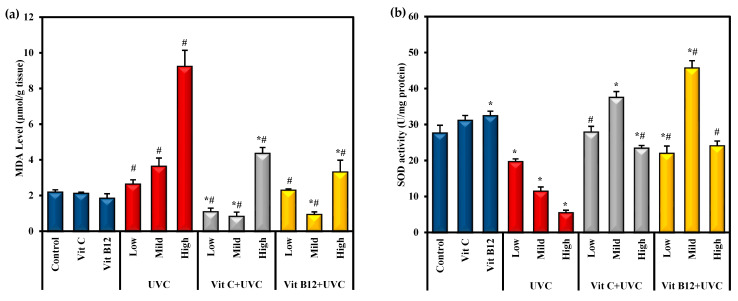

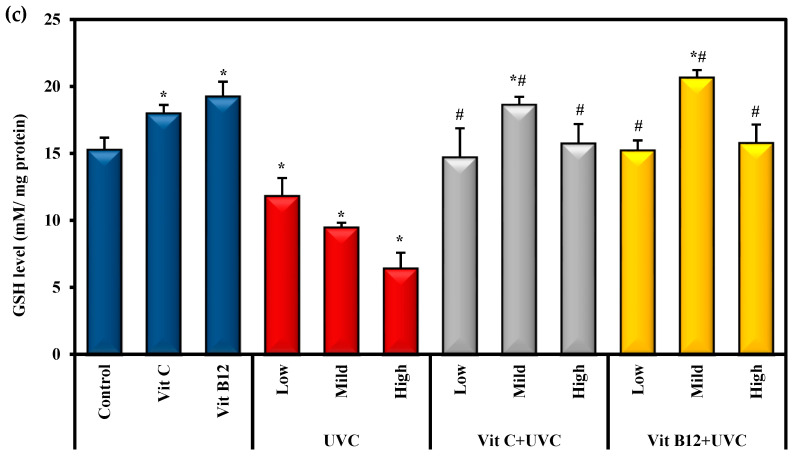
The effect of UVC irradiation, vitamin C, and vitamin B12 on hepatic oxidative stress parameters. (**a**) MDA level, (**b**) SOD activity and (**c**) GSH level. Values are provided as means ± SD of ten rats. One-way analysis of variance (ANOVA) was utilized, followed by a post hoc test (Tukey) for pairwise comparisons. *: Statistically significant compared to the control group at *p* ≤ 0.05. #: Statistically significant compared to the corresponding UVC group at *p* ≤ 0.05.

MDA was employed as an easy-to-use measure to assess the severity of lipid peroxidation reactions. The results revealed that the highest MDA level was found in groups exposed to UVC light, especially among rats exposed to high doses compared to control values (Figure 2). On the other hand, as a main index of the in vivo antioxidant activity, the GSH level and SOD activity could catalyze the scavenging of the free radical species into H_2_O. In the current study, SOD activity and GSH levels significantly (*p* ≤ 0.05) decreased upon exposure to low, mild, and high doses of UVC compared to the control. Rats exposed to a high dose of UVC showed marked significant changes in these markers compared to those exposed to low and mild doses (Figure 2). On the other hand, groups administered either vitamin C or vitamin B12 before UVC exposure showed significantly (*p* ≤ 0.05) decreased MDA levels and significantly (*p* ≤ 0.05) enhanced SOD activity, and GSH levels approached normal levels. Moreover, administering vitamin C or vitamin B12 to normal rats enhanced the antioxidant capacity, as indicated by a significant increase in SOD activity and GSH levels compared to control rats, with priority given to vitamin B12 (Figure 2).

### 2.3. The Effects of UVC Irradiation, Vitamin C, and B12 on the Hepatic Proinflammatory Cytokines

**Figure 3:**
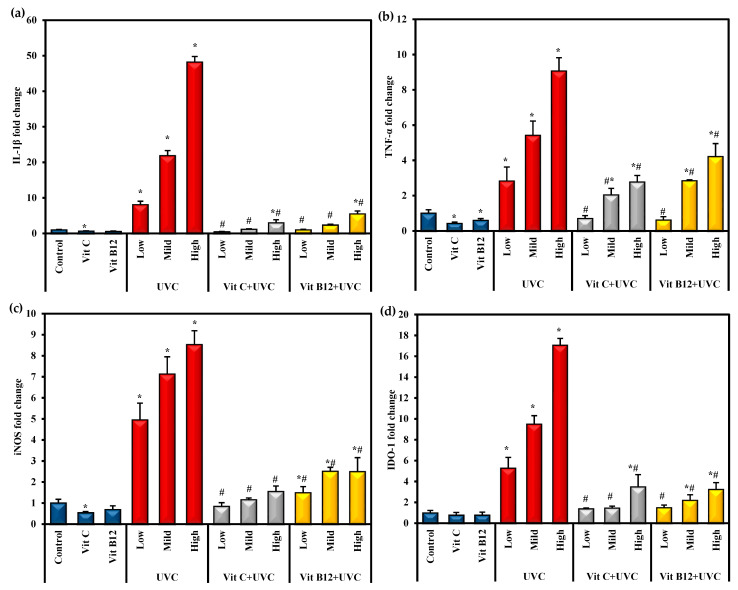
The effect of UVC irradiation, vitamin C, and B12 on hepatic IL-1β (**a**), TNF-α (**b**), iNOS (**c**), and IDO-1 (**d**) gene expression levels. Values are identified as means ± SD of four rats. One-way analysis of variance (ANOVA) was performed, then a post hoc test (Tukey) for pairwise comparisons. *: Statistically significant compared to the control group at *p* ≤ 0.05. #: Statistically significant compared to the corresponding UVC group at *p* ≤ 0.05.

Interleukin-1β (IL-1β), tumor necrosis factor-α (TNF-α), inducible nitric oxide synthase (iNOD), and indoleamine 2,3 dioxygenase-1 (IDO-1) are potent proinflammatory cytokines in systemic inflammation. The gene expression levels of these proinflammatory cytokines are illustrated in Figure 3. After exposure to UVC irradiation, IL-1β, TNF-α, iNOS, and IDO-1 gene expression levels were significantly (*p* ≤ 0.05) elevated compared with the control group. In contrast, groups administered either vitamin C or B12 before UVC exposure showed a significant (*p* ≤ 0.05) reduction in those proinflammatory cytokines compared to the animals exposed to UVC only. Moreover, administering vitamin C or B12 to normal rats showed a marked decrease in their expression levels, indicating the anti-inflammatory effects of both vitamin C and B12 (Figure 3). Generally, rats administered vitamin C showed lower levels of these proinflammatory markers than vitamin-B12-administered rats.

### 2.4. The Effects of UVC Irradiation, Vitamin C, and B12 on the Level of Cleaved Caspase-3 and DNA Fragmentation in Hepatic Tissues

**Figure 4:**
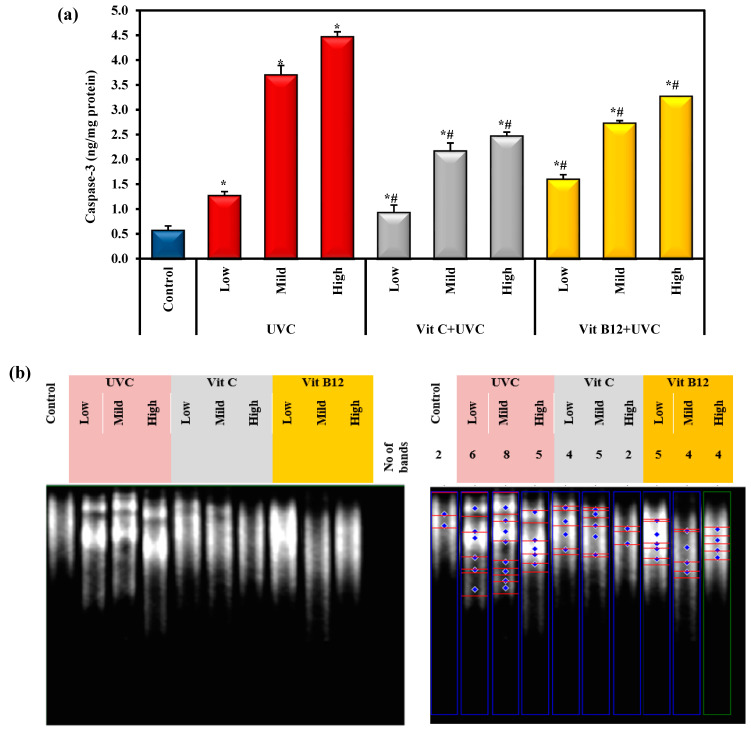
The effect of UVC irradiation, vitamin C, and B12 on (**a**) the hepatic caspase-3 level and (**b**) genomic DNA fragmentation in ethidium bromide-stained agarose gel. Blue symbols illustrate bands of DNA fragmentation. Values are given as means ± SD of four rats. One-way analysis of variance (ANOVA) was utilized, then the post hoc test (Tukey) for pairwise comparisons. *: Statistically significant compared to the control group at *p* ≤ 0.05. #: Statistically significant compared to the corresponding UVC group at *p* ≤ 0.05.

The data in Figure 4a indicate that UVC intoxication significantly elevated hepatic caspase-3 levels relative to the control level. Mild and high doses of UVC resulted in a significant increase in caspase-3 levels compared to the low UVC dose. The administration of either vitamin C or vitamin B12 significantly reduced the protein level of hepatic-activated caspase-3 compared to UVC-intoxicated groups. Additionally, the administration of vitamin C considerably reduced the level of caspase-3 and had a more significant effect than B12 administration (Figure 4a). Furthermore, these findings indicate that pre-administration of vitamin C or vitamin B12 could restrict and limit pro-apoptotic signals (caspase-3), thus offering protection. Interestingly, vitamin C administration significantly decreased caspase-3 levels compared to vitamin B12.

Figure 4b illustrates a significant genotoxic effect upon UVC light exposure compared to the control group. Meanwhile, administering vitamin C or B12 showed a significant hepatoprotective effect against UVC-light-induced DNA fragmentation. Interestingly, vitamin C showed a stronger protective effect against the high dose of UVC light than vitamin B12. Vitamin C reversed the DNA fragmentation level close to the normal value, especially at a high UVC dose (Figure 4b).

### 2.5. Histopathological Evaluations

**Figure 5:**
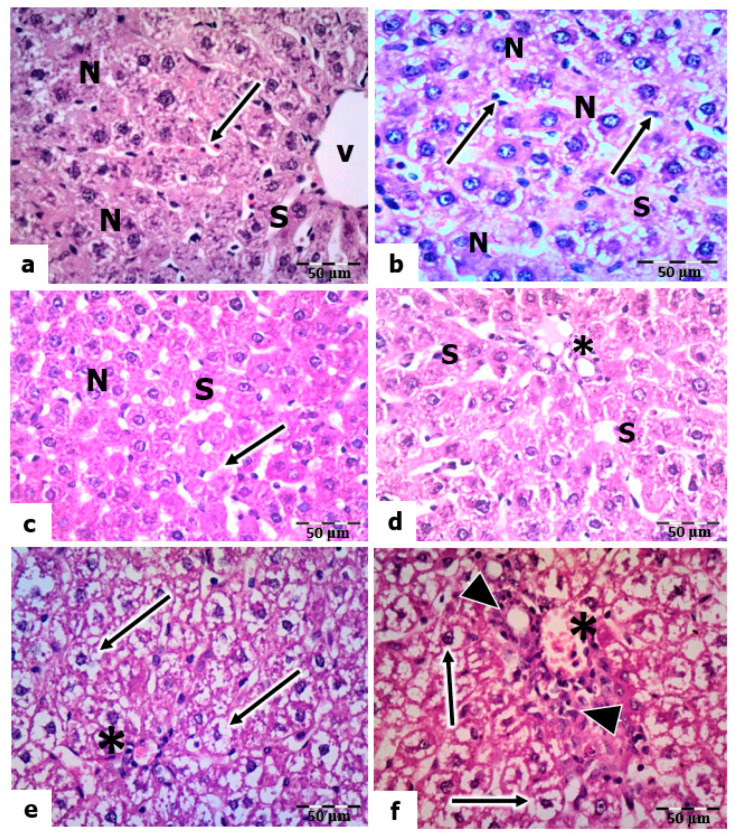
Histopathological evaluations of the hepatic tissues of control and UVC-irradiated rats. (**a**): Section of a control rat showing polygonal-shaped hepatocytes with centrally located nuclei (N), sinusoid (S), central vein (V), and Kupffer cells (arrow). (**b**,**c**): Vitamin-C- and vitamin-B12-treated rats showing normal hepatic architecture with vesicular nuclei (N) and Kupffer cells (arrows) lining the sinusoids (S). (**d**): Section of the liver of a rat exposed to a low dose of UVC showing normal nuclei (N) of hepatocytes and Kupffer cells (arrow) lining the sinusoids (S). (**e**,**f**): A section of the livers of rats exposed to mild and high UVC showed vacuolized hepatocyte cytoplasm (arrows) and congested portal vein (*); arrowheads point to hyperplasia in the wall of the bile ductulus, H&E, ×400.

The light micrographs of liver sections of the control rats stained by H& E revealed that the hepatocytes are polygonal in shape, having centrally located nuclei with prominent nucleoli and an acidophilic cytoplasm (Figure 5a). The hepatic sinusoids are lined with phagocytic Kupffer cells. In the livers of rats treated with vitamin C and vitamin B 12, the hepatocytes showed a nearly normal hepatic architecture, as in the control, where the nuclei are vesicular, and the sinusoids are lined with Kupffer cells (Figure 5b,c).

The liver sections of rats exposed to a low dose of UVC showed a normal appearance of hepatocytes (nuclei and cytoplasm). However, the sinusoids were dilated, and many Kupffer cells were observed (Figure 5d). In mild and high doses of UVC, the liver sections showed signs of degeneration, manifesting as congestion in the portal veins, hyperplasia in the bile ductulus’s wall, and the sinusoids’ dilation. In addition, the hepatocytes’ nuclei were pyknotic, and the cytoplasm was highly vacuolated (Figure 5e,f).

**Figure 6:**
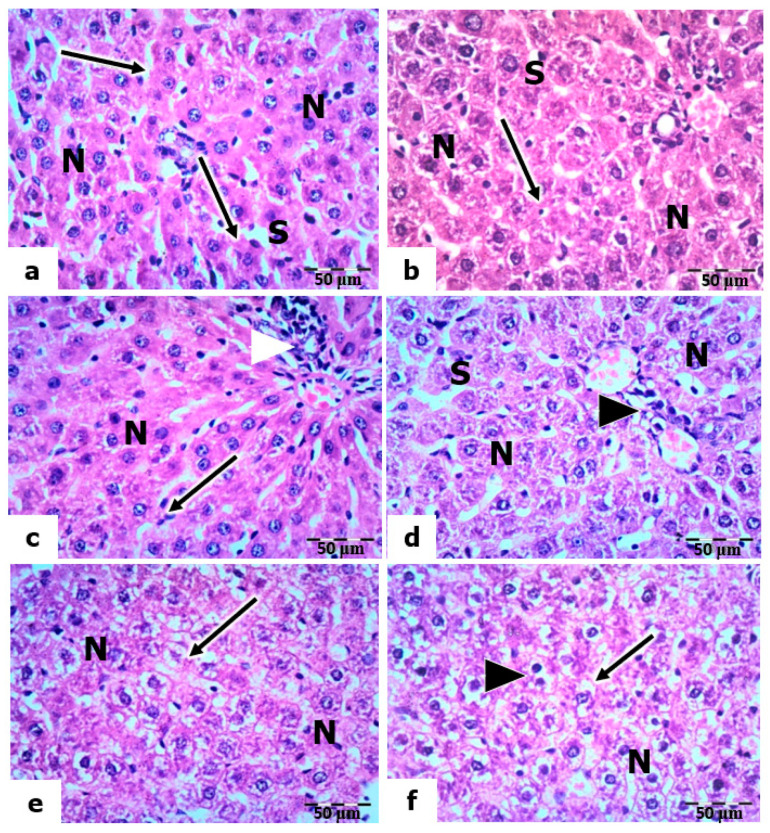
Histopathological evaluations of the hepatic tissues of vitamin-C- and vitamin-B12-pretreated rats. (**a**–**c**): Sections of the livers of rats pretreated with vitamin C and exposed to low, mild, and high UVC doses, respectively, showing normal nuclei (N) of hepatocytes and Kupffer cells (arrows) lining the sinusoids (S); arrowheads point to hyperplasia in the portal vein. (**d**–**f**): Sections of the livers of rats pretreated with vitamin B12 exposed to low, mild, and high UVC doses showing normal nuclei (N) of hepatocytes, vacuolized cytoplasm in some hepatocytes (arrows), and arrowheads pointing to bi-nucleated hepatocytes, H&E, ×400.

Meanwhile, sections of the livers of rats pretreated with vitamin C and exposed to low, mild, and high doses of UVC showed a noticeable reduction in structural abnormalities compared to those of UVC-exposed rats. The hepatocytes are normal in their polygonal-shaped appearance, having centrally located vesicular nuclei, and the cytoplasm is acidophilic. In addition, many activated Kupffer cells were observed along the narrow sinusoidal spaces (Figure 6a–c). However, certain hyperplasia in the wall of the bile ductile is still observed in sections exposed to high-dose UVC (Figure 6c). Furthermore, the light micrographs of liver sections of rats pretreated with vitamin B12 and exposed to low, medium, and high UVC doses showed the appearance of normal hepatic architecture, as in the control; the nuclei of hepatocytes are vesicular, and their nucleoli are prominent. However, the cytoplasm of a few hepatocytes was vacuolized (Figure 6d–f).

### 2.6. Electron Microscopic Observation

**Figure 7:**
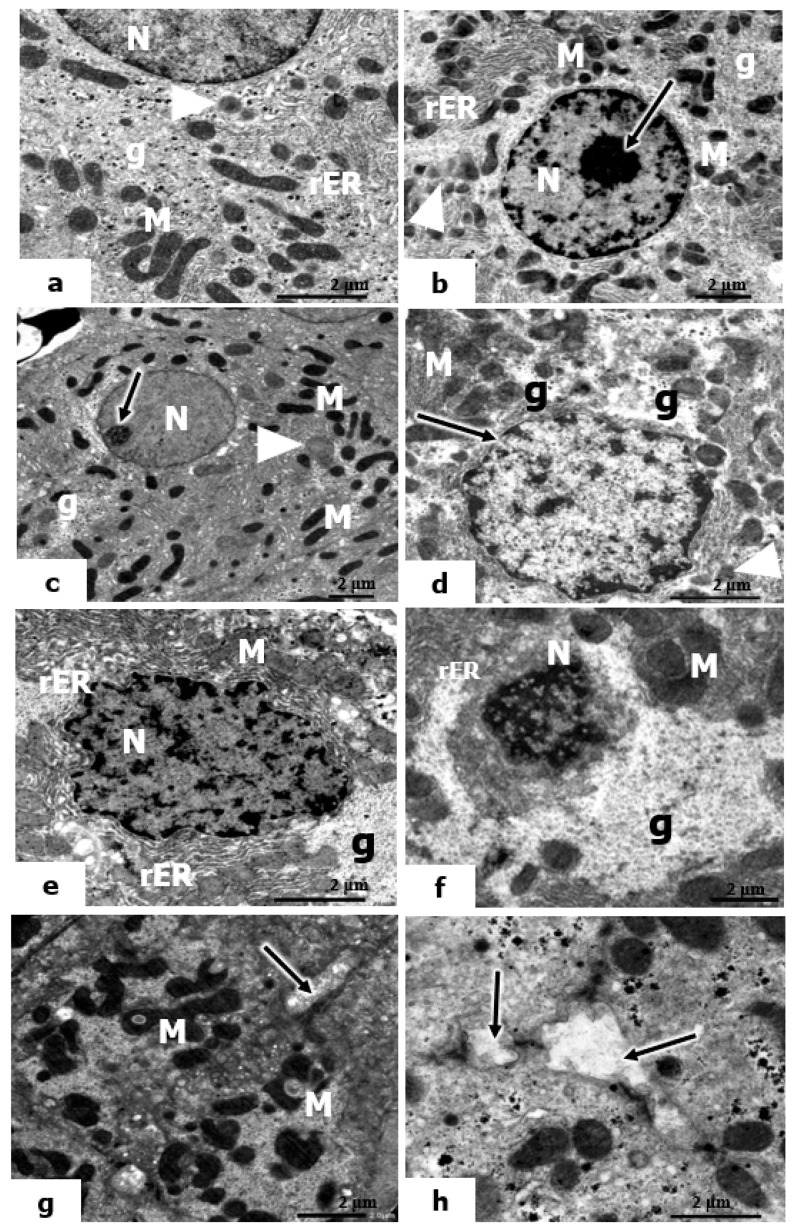
Electron micrographs of the hepatic tissues of control and UVC-irradiated rats. (**a**–**c**): Electron micrographs of liver sections of control rats and rats treated with vitamin C and vitamin B12, showing sections of rounded-shaped nuclei (N) of hepatocytes, nucleoli, mitochondria (M), cisternae of the rough endoplasmic reticulum (rER), glycogen particles (g), and small lysosomes (arrowheads). (**d**): Electron micrograph of livers of rats exposed to low UVC showing an irregular nuclear outline of the nuclear envelope nucleus (arrow), mitochondria (M), glycogen content (g); the arrowhead points to small lysosomes; (**e**–**h**): Electron micrographs of livers of rats exposed to mild and high UVC showing pyknotic nuclei (N), vacuolized mitochondria (M), glycogen content (g), short membranes of rER; microvilli in the bile canaliculi (↑) (×10,000).

The electron micrographs of control rats’ livers and those administered vitamin C or B12 (Figure 7a–c) showed the appearance of normal-shaped nuclei surrounded by a well-developed nuclear envelope. The cytoplasm contains scattered mitochondria, short, flattened cisternae of rough endoplasmic reticulum (rER), glycogen particles, and a few lysosomes.

In the ultrastructure appearance of hepatocytes in the livers of rats exposed to low-dose UVC, the hepatocyte nucleus exhibited little irregularity in its nuclear envelope. The cytoplasm contains mitochondria, a few lysosomes, and glycogen particles (Figure 7d). However, the hepatocytes in the livers of rats exposed to mild and high doses of UVC showed remarkable injury in both the nucleus and cytoplasm. The hepatocytes exhibited marked irregularities in their nuclear envelope, and the heterochromatin was condensed (a sign of pyknosis) (Figure 7e–h). In the cytoplasm of both mild and high UVC doses, the mitochondria were swollen, having indistinct cristae; they were pleomorphic in shape, and most of them were vacuolized (Figure 7e,g). The rER appeared as significantly dilated and interrupted cisternae. In addition, there was a marked increase in the glycogen content displacing the organelles to the periphery, and the microvilli in the bile canaliculi were lost (Figure 7e,f,h).

**Figure 8:**
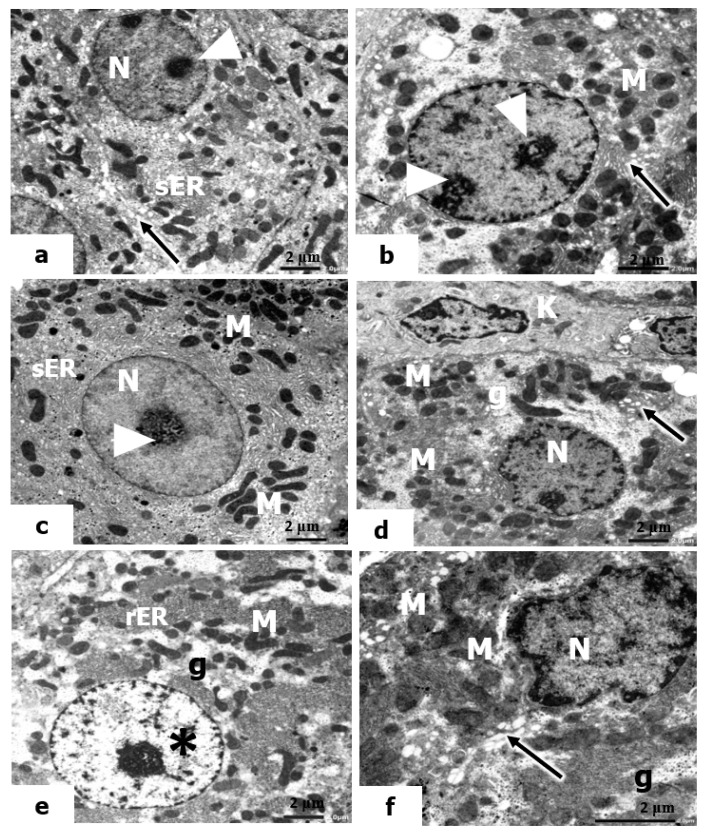
Electron micrographs of livers of rats pretreated with vitamin C and vitamin B12 and exposed to low, mild, and high doses of UVC. (**a**–**f**): The nuclei (N) of hepatocytes that maintain their normal cellular boundaries, euchromatic nucleus (*), large-sized nucleoli (arrowheads), mitochondria (M), short, flattened cisternae of rER, Kupffer cell (K), active Golgi bodies (arrows), proliferated smooth ER (sER), and small areas of glycogen (g) (×10,000).

We also considered the ultrastructure appearance of hepatocytes in the livers of rats pretreated with vitamin C and exposed to low, mild, and high doses of UVC. The hepatocytes showed certain amelioration in the hepatic structure, which seemed to be normal tissue (Figure 8a–c). The nuclei of hepatocytes contain large, actively proliferated nucleoli, and the mitochondria are numerous in the cytoplasm. In addition, the Golgi body increased, and the smooth ER (sER) increased significantly, indicating its role in detoxification.

Furthermore, in the electron micrographs in the liver sections of rats pretreated with vitamin B12 and exposed to low, mild, and high doses of UVC, the hepatocytes revealed a significant resemblance to the control, where they maintained their normal cellular boundaries. The nuclei have prominent nucleoli and distinct nuclear envelopes. The cytoplasm contains many mitochondria; small areas of glycogen content and the Golgi bodies were actively observed (Figure 8d–f). It is worth noting that treatment with vitamin B12 as a prophylactic agent against the hazardous effects of UVC was apparent in rats exposed to low and mid doses of UVC to a greater extent than in those exposed to high doses.

## 3. Discussion

Ultraviolet radiation is one of the most critical abiotic factors for life on Earth. Despite its benefits, sunlight threatens living organisms, and excessive UV radiation of certain wavelengths can promote carcinogenesis with well-known genotoxic and mutagenic effects [49,50]. Solar UV radiation can be subdivided into UVA, UVB, and UVC components. However, because the ozone layer absorbs the vast majority of UVC, ambient sunlight is predominantly UVA (90–95%) and UVB (5–10%). Humans are exposed to UVC through artificial sources such as fluorescent lamps or lasers, mercury vapor lamps, and light-emitting diodes [15]. UV penetrates the skin in a wavelength-dependent manner. The skin’s ability to absorb UV radiation decreases rapidly with decreasing wavelength. UVA (Longer wavelength) penetrates deeply into the epidermis reaching well into the dermis. In contrast, UVB (medium wavelength) is almost completely absorbed by the epidermis, with comparatively little reaching the dermis. DNA damage from UV irradiation may occur due to UV’s direct and indirect effects. Direct damage is related to the covalent modification of neighboring pyrimidines, whereas indirect damage is related to ROS accumulation [51].

UVA’s detrimental effect on DNA is associated with excessive ROS formation that induces oxidative DNA damage via indirect photosensitizing reactions [52]. UVB and UVC (shorter wavelength) are directly absorbed by DNA-forming photo-dimers at di-pyrimidine sites [14,15,51]. It has also been reported that UVC light stimulates ROS production, which has the potential to directly damage DNA and other macromolecules [50]. Mutations and cancer can result from many of these modifications to DNA.

UVC exposure has become a crucial risk for living organisms due to its widespread use in sterilization and food preservation processes [52]. In addition, the necessity of placing the UVC unit very close to the target was also considered a disadvantage. The severe damaging effect of UVC arises from the fact that the wavelength of 254 nm, which is often used in sterilization, is very close to the peak of DNA absorption (253.7 nm) and can effectively eliminate microorganisms such as bacteria, fungi, viruses, and spores. Despite its important and numerous applications, UVC radiation is cytotoxic, detrimental to the human cornea, and promotes the development of malignant tumors [14,52]. UV radiation is a critical factor in the onset and progression of a variety of skin disorders. Interestingly, “non-skin” tissues and internal organs can be negatively influenced by UV radiation through low-molecular-weight mediators formed upon irradiation [53]. The current study aimed to evaluate the hepatotoxicity induced by UVC irradiation and the possible hepatoprotective effects of vitamin C and B12 on the detrimental changes induced by UVC radiation of the skin and subsequent events leading to the injury of the rats’ liver cells.

This experiment applied UVC radiation to rats for 14 days with 30 W UVC lamps (254 nm and 0.0014 J/cm^2^). These doses of UVC radiation resulted in functional and structural alterations in the liver cells of rats. UVC-irradiated rats showed a marked elevation in serum ALT and AST activities, associated with a significant decline in the serum levels of total proteins and albumin compared to the control group, which suggested the presence of significant liver damage (hepatotoxicity). The elevation of serum transaminases was likely due to hepatocyte injury that increases the cell membrane permeability, leading to the leakage of these enzymes into circulation after UVC exposure [54]. Similar findings were previously reported after exposure to gamma rays [54,55] and confirmed radiation-induced hepatic damage.

Furthermore, whole-body UVC irradiation revealed hyperlipidemia, as represented by a significant elevation in the serum total cholesterol and triglyceride levels upon increasing the UVC irradiation doses compared to the control rats. Radiation-induced hyperlipidemia may be attributed to the alterations in hepatic lipid metabolism and may result from radiation’s indirect effect on the release of several inflammatory mediators [56,57]. Various inflammatory mediators consist of modified lipids, such as oxidized LDL-cholesterol. Moreover, chemokines, cytokines, and lipid mediators further foster endothelial dysfunction and increase vascular permeability [58]. Generally, the ionizing radiation was found to increase endothelial cells’ permeability, further stimulating the influx and accumulation of lipids and immune cells in the intimal layer of the vascular wall, resulting in a vicious circle [59]. This explains the interrelation and the crosstalk between hyperlipidemia and the inflammatory process. Radiation-induced hyperlipidemia and hepatotoxicity are commonly reported post-gamma-ray exposure rather than UVC exposure [54].

Oxidative stress is essential for developing hepatic degeneration and fibrosis [60,61]. Ionizing radiation induces oxidative stress on target tissues by increasing the generation of ROS, which causes an imbalance between cellular pro-oxidant and antioxidant defenses [62]. ROS attacks cellular macromolecules, including nucleic acids, lipids, and proteins, ultimately leading to cell death. Peroxyl radicals are produced when ROS attack polyunsaturated fatty acid residues. These radicals undergo a cyclization process to endoperoxides, producing 4-hydroxynonenal and MDA [13]. MDA is the most important product of lipid oxidation [61]. Lipid peroxidation is one of the molecular mechanisms involved in UVC irradiation cytotoxicity [63]. The body has many antioxidant agents that it uses as a defense mechanism and can counteract the damage caused by free radicals [19,20]. GSH is vital to cellular antioxidant defenses. GSH directly scavenges free radicals and prevents the oxidation of -SH groups in proteins. Indirectly, it is a necessary cofactor for the antioxidant enzymes GPx and GST, which detoxify lipid peroxides, H_2_O_2_, and electrophiles [18,60]. GSH endorses an elevation of sensitivity to oxidative damage, indicating an inverse relationship between lipid peroxidation and the GSH level. In addition, SOD constitutes a highly important antioxidant defense against oxidative stress in the body. SOD is the front line of defense against superoxide radicals, catalyzing the radical’s rapid dismutation into H_2_O_2_ and molecular oxygen (O_2_) [13,61].

One of the major impacts of radiation on irradiated tissues is the suppression of antioxidant enzymes, which leads to the generation and accumulation of ROS and subsequently increases lipid peroxidation and tissue damage [64,65]. In the current study, measuring the levels of MDA and GSH and monitoring the activity of the major antioxidants (SOD) allowed for a better understanding of the metabolic processes underlying liver injury caused by UVC radiation. The data indicated that UVC irradiation induced a significant elevation in MDA levels associated with a significant reduction in the specific activity of the antioxidant enzyme SOD and the non-enzymatic antioxidant (GSH) content in the liver tissues. The decrease in the activities of antioxidant enzymes is a consequence of increased oxidative stress [13]. UVC-mediated oxidative stress was previously reported in various models [63,66]. Goswami and Sharma [63] reflected elevated MDA levels accompanied by a depleted SOD activity following UVC exposure and indicated the occurrence of ROS-mediated protein denaturation, enzyme inactivation, and cellular damage.

Oxidative stress and inflammation are the major and primary interdependent mechanisms that contribute to the development of hepatic fibrosis and damage to the liver. In addition to lipid peroxidation, oxidative stress upregulates the expression of proinflammatory cytokines such as IL-1β and TNF-α, indicating the interplay between oxidative stress and inflammation [67,68]. Proinflammatory mediators such as IL-1β, IL-6, and TNF-α are messengers for the upregulation of the inflammatory cascade. Radiation exposure was reported to affect TNF-α, IL-1β, IL-6, IL-12, and type I interferon expression levels. It has been confirmed that the accumulation of inflammatory cells following radiation exposure mediates normal tissue destruction through various signaling pathways [69,70]. The significant elevation in the expression levels of hepatic TNF-α, IL-1β, and iNOS confirms these findings. TNF-α is a hallmark of inflammation. The elevated TNF-α level may be related to the increased ROS production. This has been demonstrated to upregulate the iNOS, which subsequently increases the production of the proinflammatory mediator nitric oxide [71]. Therefore, the suppression of proinflammatory cytokines has been studied to alleviate radiation injury in various irradiated tissues [72,73,74].

IDO-1 is a cytosolic heme-containing enzyme that catalyzes tryptophan catabolism and induces hypoxia [75]. IDO is overexpressed in response to oxidative stress as well as inflammation. Studies have reported that inflammatory cytokines (TNF-α and IL-1β) upregulate IDO-1 expression [76,77,78]. The current study suggested a link between increased inflammatory cytokines (TNF-α, IL-1β, and iNOS) and IDO1 expression levels with UVC-induced oxidative stress. A study by Nagalaxmi and Praveen Kumar [64] indicated that UVB irradiation for 12 h and 24 h decreased GSH, increased carbonyls and MDA levels, and elevated IDO-1 activity, reflecting the correlation and protective role of IDO-1 in UVB- induced oxidative stress.

Furthermore, ROS production is important in the UVC induction of DNA adducts in carcinogenesis and apoptosis initiation. Therefore, genomic damage induced by UVC is primarily expressed through oxidative DNA adducts [4,16]. Chemicals and other environmental factors continually attack the human body, potentially harming DNA through both oxidative and non-oxidative mechanisms. Oxidative stress, genotoxicity, and apoptosis are closely related [79]. The current results showed a significant increase in cleaved caspase-3 levels in the liver tissue of UVC-irradiated rats. Caspase-3 is one of the best-known essential downstream apoptotic initiators that triggers caspase-activated DNase, an active endonuclease that cleaves DNA during apoptosis and results in DNA fragmentation [80]. Generally, activated apoptosis may be linked to the overproduction of ROS, which induces a significant elevation in activated caspase-3 levels, leading to the apoptotic condition.

Moreover, the present results show that UVC-induced apoptosis was confirmed via the induction of DNA fragmentation, where its level was significantly elevated relative to the control level. DNA fragmentation is considered a prominent hallmark of apoptosis [80]. In keeping with our findings, Shih and Cherng [16] and Begović and Antunovic [81] reported that UVC radiation-induced apoptosis directly through the alteration of caspase-3 activity, FAS-associated protein with death domain (FADD), and poly (ADP-ribose) polymerase (PARP) protein cleavage, which can explain the mechanisms of DNA damage. Moreover, high levels of ROS can directly cause damage to nuclear and mitochondrial DNA and activate the three main pathways of apoptosis: Mitochondrial, death receptor, and endoplasmic reticulum [82,83]. Mitochondrial intrinsic apoptosis was activated via upregulation of p53 and JNK signaling, which activates pro-apoptotic Bcl-2 proteins (Bak, Bax, Bad, Puma, etc.) that block the anti-apoptotic activity of Bcl-2 and Bcl-XL. Moreover, ROS causes mitochondrial membrane depolarization and/or the opening of Bax/Bak channels, followed by the formation of an apoptosome complex, which activates caspase-9. Caspase-9 then activates effector caspases such as caspase-3–6 and -7, resulting in the cleavage of cellular proteins and cell death by apoptosis [83]. The death receptor extrinsic apoptotic pathway was activated by ROS via recruitment of the adaptor protein FAS-associated protein with death domain (FADD) among others and procaspase-8 or -10 to the cytoplasmic surface of the receptor to form the death-inducing signaling complex (DISC). This results in the activation of caspases-8/-10, which in turn will activate executioner caspases-3 and trigger apoptosis [84]. Moreover, ROS stimulates the endoplasmic reticulum apoptotic pathway through the activation of endoplasmic reticulum stress sensors, which finally activates several caspases, such as caspase-3/-4/-7/-8/-9 and -12, which eventually engages the apoptotic program [82]. As a result, UVC could modulate the intracellular signal transduction pathways by activating oxidative stress, subsequently enhancing inflammatory and apoptotic reactions.

Defense against oxidative stress and inflammatory reactions is sustained using numerous mechanisms, including antioxidant machinery. Exogenous supplementation of antioxidants has been found to reduce UV-induced oxidative damage. However, it is unclear how these antioxidants affect the components of signaling pathways [85]. Although there is no approved direct influence of vitamin C and B12 against UVC intoxication, these vitamins have been developed to combat “oxidative stress” [86]. Recent trials suggest that various emerging strategies using antioxidants and anti-inflammatory plant-derived materials may be used as protective agents against UV rays [85]. The current study indicates that vitamins C and B12 alleviated liver enzyme activities and normalized albumin, total protein, total cholesterol, and triglycerides levels. Vitamin C and B12 offered hepatoprotective effects by restoring the hepatic antioxidative enzyme activities (particularly SOD activity), reducing lipid peroxidation (MDA level), and increasing the GSH level. Vitamin C’s hepatoprotective effect was previously reported against radiation-induced hepatotoxicity through its antioxidant properties [87]. Vitamin C has strong antioxidant properties; it can directly capture O_2_^•−^ and OH^•^ and prevent lipid oxidation. It interacts with small-molecule antioxidants, such as GSH, tocopherol, and coenzyme Q. Vitamin C was reported to enhance the production and activation of antioxidant enzymes, including SOD, GPx, or catalase. Moreover, Vitamin C improves the activity of transcription factors, which permits the expression of genes responsible for producing antioxidant proteins [30,88]. Vitamin B12 is an essential micronutrient that directly and indirectly reduces oxidative damage. B12 directly scavenges ROS. It indirectly boosts ROS scavenging by preserving GSH levels, modulating cytokine and growth factor production, reducing homocysteine-induced oxidative stress, and reducing advanced glycation end-product-induced stress [41,45]. In this regard, administering vitamin C or B12 protects DNA, proteins, and lipids from UVC-induced oxidative reactions.

In addition, the data presented here confirm the significant role of vitamins C and B12 in regulating the inflammatory response. Vitamin C and B12 significantly decreased the gene expression levels of TNF-α, IL-1β, iNOS, and IDO-1 in the hepatic tissue of UVC- irradiated rats. Most of them were normalized to the control levels. These findings support vitamins’ previously stated anti-inflammatory action [30,89,90,91]. Previous studies illustrated the ability of vitamins to reduce the inflammatory biomarkers in the liver tissues of rats, either through a direct effect on their expression levels or through the mitigation of oxidative stress [88,91,92,93]. Vitamin B12 is a necessary cofactor for methionine synthase, the enzyme responsible for successful methionine regeneration from homocysteine. Methionine is essential for the universal methyl donor S-adenosyl-L-methionine (SAM), which is required for the epigenetic methylation of histones and DNA that regulate gene expression and play roles in human health and disease [25,94,95]. In addition, vitamin C selectively influences intracytoplasmic cytokine production. The mechanism underlying the anti-inflammatory effects of Vitamin C is tightly related to its antioxidant activity [25,30]. Similar effects were reported by γ-radiation and natural antioxidants. Alkhalf and Khalifa [62] elucidated the role of blueberry extract antioxidants in mitigating γ-radiation-induced hepatic damage by restoring liver pro-oxidant status, reducing cytokine levels (IL-6, IL-10, and TNF-α), ameliorating hepatic histopathological alterations, and reducing DNA damage.

The major molecular target for UV radiation is DNA. The ionizing radiation can cause DNA lesions by direct deposition of energy in the DNA and by the indirect action through reactive chemical species (mostly free radicals) formed by the radiolysis of water molecules near the DNA [96]. These free radicals are converted to ROS and can also generate reactive nitrogen species (RNS) through the upregulation of several enzymes, including iNOS. ROS and RNS can attack the DNA resulting in several alterations, including DNA single-strand and double-strand breaks, base damage, and the destruction of sugars. Moreover, ionizing radiation can initiate a reaction between two molecules of thymine, one of the bases that make up DNA [64,97]. Abou-Zeid and El-Bialy [54] reported that the significant DNA fragmentation in irradiated rat tissues, and the associated DNA lesions, were most likely generated by ROS, which has previously been demonstrated to cause DNA strand breaks and cross-link proteins and DNA. The improvement of DNA following the pretreatment with vitamins and such enhancement may be due to the effect of vitamin C in activating the DNA repair system and enhancing protein synthesis [98,99,100]. In addition, vitamin B12 is necessary for the methylation process, which is important in DNA and protein synthesis and is essential for cellular energy production [101,102]. Hence, vitamins C and B12 are of great interest in maintaining genomic stability. Our results suggest the antiapoptotic action of vitamins C and B12 through a significant reduction in activated caspase-3 levels and DNA fragmentation in irradiated rats pretreated with vitamins (C and B12). These findings concur with the previous reports of Rössig and Hoffmann [103], Hassan and Salem [104], and Gęgotek and Ambrożewicz [105], indicating the antiapoptotic action of vitamin C; moreover, Majumdar and Maiti [106] and Wu and Xu [107] illustrated the antiapoptotic efficacy of vitamin B12. Interestingly, vitamin administration could decrease UVC-induced hepatic dysfunction in rats. This occurs through its anti-lipid peroxidative, anti-inflammatory, and antiapoptotic efficiency (i.e., by decreasing the levels of MDA and the depletion of TNF-α, IL-1β, iNOS, IDO-1, and caspase-3 levels in the tissues).

Finally, the biochemical results were further confirmed by the hepatic degeneration and activation of Kupffer cells that mediate UVC-induced oxidative damage. The current results declared many activated Kupffer cells in the liver tissues of rats exposed to high-dose UV radiation, indicating their important role in defense against radiation. In the liver tissue, the Kupffer cells constitute approximately 20% of all hepatic cells [108]. Kupffer cells are resident liver macrophages and constitute 80–90% of total tissue macrophages in the body. Kupffer cells play crucial roles in phagocytosis, antigen presentation, and cytokine synthesis during inflammation [109]. The activation of Kupffer cells promotes the release of inflammatory mediators, growth factors, and ROS [110]. This activation is essential for the liver’s normal physiological function, such as its response to infection or injury [111].

The present results showed no aberrant liver damage in the control tissues. However, rats exposed to all doses of UVC radiation for 14 days, 8 h daily, showed marked histopathological and ultrastructural alterations in hepatocytes. The hepatocytes assumed a spongy appearance. In addition, the nuclei of these hepatocytes showed significant deterioration, which increased gradually with the increased dosage used for exposure. Several studies have shown that long-term exposure to UV radiation can potentially harm interior organs such as liver tissue [53,112]. Wiedemann and Manfredi [113] explained that hepatocytes’ cytoplasms might become vacuolated due to salt and water retention, which causes cellular swelling.

Similarly to the hazardous effects of UV radiation (UVA + UVB) on the liver tissue, the current results for UVC-irradiated rats follow the results of Keskin and Acikgoz [114], who found hepatocyte enlargement and bile duct proliferation. The study of Yel and Türker [115] found that UVC irradiation for 52, 112, and 168 h revealed marked harmful alterations to the stratum basale of mole rats’ epidermis. The effect depended on the exposure time and radiation dosage applied. Furthermore, in the lung cells of mole rats, UVC radiation caused many signs of necrosis in the alveolar epithelial cells, and the zymogen granules of the pancreatic exocrine cells were decreased in mole rats [116,117].

Moreover, the current results are in accordance with the previous report of Tekın and Türker [118], who observed significant ultrastructural alterations in the liver tissue, including a decrease in cytoplasmic organelles, dilatation in rER, damage to the nucleus membrane, and widened and vacuolated mitochondria in the cytoplasm after 7, 14, and 21 days of UVC irradiation. The present results showed that the presence of pyknotic nuclei of the hepatocytes, swelling and vacuolation of mitochondria, and dilatation of rER in the cytoplasm were the most observable alterations in the hepatocytes of UVC-irradiated rats. Exposure of liver cells to UVC radiation caused the most obvious alterations in the mitochondria and rER. Alterations in the permeability of mitochondrial membranes may be the cause of vacuolation. Radiation investigations, including those using X-rays and electromagnetic fields, confirmed the presence of irregularly shaped mitochondria, fragmented rER, widely dispersed ribosomes, a vacuolated cytoplasm, and electron-dense bodies, all of which are consistent with the aforementioned findings [119].

These degenerative changes would disrupt the structure of cells and reduce their function. On the other hand, vitamin C and B12 were found to improve all of these changes in liver structure, although vitamin C was more effective than vitamin B12. This is supported by the findings of Hassan and Salem [104] and Su and Liang [91], indicating the potential hepatoprotective effect of vitamin C, and Ahmad and Afroz [93] and Majumdar and Maiti [106], which illustrate the hepatoprotective action of vitamin B12.

## 4. Materials and Methods

### 4.1. Chemicals and Reagents

The analytical-grade vitamins, reagents, and chemicals were obtained from Sigma-Aldrich Chemical Co. (St. Louis, MO, USA). Kits for aminotransferases, total protein, and albumin were purchased from Biodiagnostic (Giza, Egypt). Kits for the lipid profile were obtained from Diamond Diagnostics (Cairo, Egypt). Primers were obtained from Willowfort (Nottingham, UK).

### 4.2. Irradiation Systems: UVC Source and Lamps Characteristics

UVC lamps (40 watts and 45 cm in length), model F40W/2 FT/T12/6 L 368, made in Germany, were placed 30 cm above the cover of the rat’s cage. The UVC lamps were calibrated in the National Institute of Standards—NIS Radiometry Metrology Lab. Reference Radiometry S480/268-UVC—Report No.79/52/2022, Cairo, Egypt. UVC lamp calibration is illustrated in the supplementary materials. The experimental setup involves a timer and a power supply controller. The timer managed the UV exposure time (8 h/day). The power supply controller checks and controls the power supply’s stability during exposure. It gives a measure for the whole exposure time over the experimental period. A one-hour feeding break was provided at midday. The average exposure doses to UVC radiation, with daily interval exposure of 8 h for 14 days, were measured to be 725.76, 967.68, and 1048.36 J/cm^2^, representing low, mild, and high doses, respectively. The UV light lamps were measured to have a wavelength of 254 nm, and an energy of 0.0014 J/cm^2^ was emitted in 1 s.

### 4.3. Experimental Design and Sample Collection

In total, 120 male adult Sprague Dawley rats (weighing 180–200 g) were purchased from the animal’s house of the Faculty of Medicine, Alexandria University, Alexandria, Egypt. Rats were acclimatized for 2 weeks and maintained on a basal diet with free access to running water under controlled environmental conditions at room temperature 25 ± 2 °C with humidity 50 ± 10%, at a normal day/night photoperiod. All animals’ experimental procedures followed the accepted guidelines for the Institutional Animal Care and Use Committee (IACUC), Alexandria University, AU 04201111102 (2020). The study was carried out following the ARRIVE guidelines.

**Figure 9:**
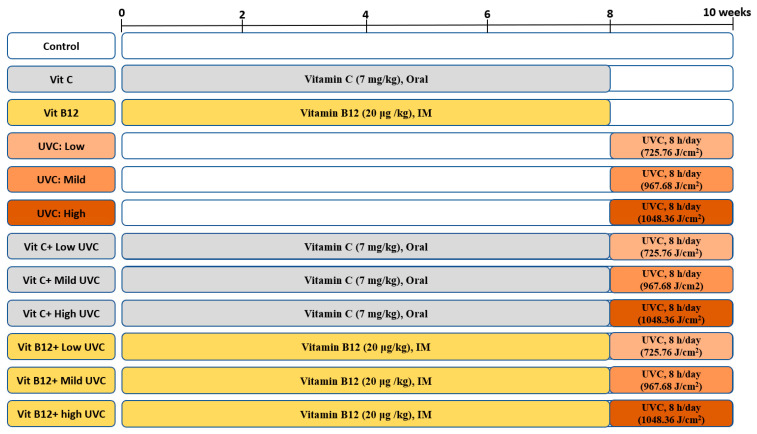
An illustration of the experimental design groups.

UVC-irradiated rats were exposed to low, mild, and high doses of artificial UVC light, for eight hours per day, for the last two weeks of the experiment. Before UVC exposure, rats were anesthetized, and the dorsal skin was shaved (Figure S1). Vitamin C was administered daily at a dose of 7 mg/kg BW orally by gavage for 8 weeks [120,121]. In comparison, vitamin B12 (hydroxocobalamin) was injected intramuscularly (IM) day by day at a dose of 20 μg/kg BW during the protection period (8 weeks) [122]. Rats were split into 12 groups (10/group) according to UVC dose and treatment, as illustrated in Figure 9. Rats exposed to UVC radiation showed a slight increase in body temperature (only during the exposure time) with no significant signs of skin damage.

Ultimately, rats were fasted overnight and euthanized under isoflurane inhalation. Serum was extracted from blood samples and stored at −20 °C until it was needed for various biochemical studies. Small pieces of the right lobe of the liver tissue were excised carefully and fixed for histopathological and electron microscopic investigations. Another liver portion was stored at −80 °C for biochemical, RT-PCR, and DNA fragmentation analysis.

All the biochemical procedures were applied with maximum attention. They were performed by the same operator, using the same instrument and the reagents were prepared once for each parameter that was measured for all groups at the same time to avoid any change in handling, preparations and instrument calibration.

### 4.4. Biochemical Investigations

#### 4.4.1. Serum Liver Function Indicators and Lipid Profile

Using commercially available kits from Biodiagnostic, Egypt, the liver markers AST, ALT, total protein, and albumin levels were measured in the serum. Lipid profile indicators, such as serum total cholesterol and triglycerides levels, were measured using commercial kits from Diamond Diagnostics.

#### 4.4.2. Preparation of the Liver Homogenates and Oxidative Stress Parameters

The liver tissues were homogenized (10%, *w/v*) in ice-cold phosphate-buffered saline (0.1 M, pH 7.4). The homogenate was centrifuged at 10,000× *g* for 20 min at 4 °C. The collected supernatant was frozen at −20 °C until it was assayed to assess lipid peroxidation biomarkers, SOD activity, and the reduced glutathione level.

**Malondialdehyde (MDA),** one of the aldehyde products of lipid peroxidation, was measured in the liver tissues as a calorimetric indicator of lipid peroxidation. The thiobarbituric acid (TBA) spectrophotometric method was used. MDA reacts with TBA in an acidic environment producing a pink-colored product upon heating, which can be detected spectrophotometrically at 532 nm [123]. The level of hepatic MDA was quantified as μmol/g tissue.

**Superoxide dismutase (SOD)** activity was estimated in liver tissue using pyrogallol as a substrate [124]. The amount of enzyme that inhibited the pyrogallol autooxidation by 50% was considered one unit of SOD activity. U/mg protein units were used to express SOD activity.

**Reduced glutathione (GSH)** levels were assessed using Ellman [125]. GSH reacts with 5,5′-dithiobis (2-nitrobenzoic acid) (Ellman reagent, DTNB), generating a yellow-colored product that was monitored at 412 nm. The level of GSH was given as mM/mg protein.

### 4.5. Total RNA Extraction and Quantitative Real-Time PCR Analysis

According to the manufacturer’s instructions, total RNA was extracted from liver tissues (approximately 100 mg) using GENEzol™ Reagent (Geneaid, Taipei, Taiwan). Using a NanoDrop 2000 spectrophotometer (Thermo Scientific, Waltham, MA, USA), the RNA concentration and purity were assessed. cDNA was synthesized using TOPscriptTM RT DryMIX (dT18/dN6) (Enzynomics, Daejeon, Republic of Korea) following the manufacturer’s instructions. Primers’ sequences for inflammatory markers are shown in Table 1.

The PCR reactions contained 10 µL of TOPreal™ qPCR 2X PreMIX (SYBR Green with low ROX) (Enzynomics, Republic of Korea), 1 µL of cDNA, 1µL of each primer, and RNase-free water in a total volume of 20 µL. PCR was conducted using the following thermal cycler protocol: Initial denaturation by pre-incubation at 95 °C for 12 min, cDNA was amplified for 40 cycles of denaturation at 95 °C for 15 s, annealing for 30 s as mentioned in Table 1, and extension at 72 °C for 30 s. PCR amplification and analysis were achieved using a CFX96™ Real-Time System (BIO-RAD, Hercules, CA, USA). The target gene’s critical threshold (Ct) was used to calculate the fold change using the 2^−∆∆Ct^ method. The housekeeping gene GAPDH was used to normalize the data.

### 4.6. Cleaved Caspase-3 Level and DNA Fragmentation

Cleaved-caspase-3 levels were evaluated using a RayBio^®^ Human/Mouse Cleaved-Caspase-3 (Asp175) ELISA kit (RayBiotech Life, Inc., Norcross, GA, USA) following the kit manufacturer’s instructions. Cleaved-caspase-3 levels are expressed in ng/mL.

To evaluate the genotoxicity of different treatments, DNA fragmentation was applied. The total genomic DNA was isolated from the liver tissue samples via the protocol illustrated in E.Z.N.A.^®^ Tissue DNA Kit (D3396-01, Omega Bio-TEK, Norcross, GA, USA). Then, 15 μg of DNA/lane was loaded on agarose gel (1.5%, *w*/*v*), stained with Ethidium bromide (10 μg/mL), and separated by electrophoresis (75 V, 150 mA). Trans-illumination with ultraviolet light (Consort, Turnhout, Belgium) was used to visualize DNA bands. The gel documentation system (GelDoc-It, UVP, Cambridge, UK) was adapted for data analysis using Totallab analysis software, ww.totallab.com (Ver.1.0.1).

### 4.7. Histopathological Study

Small pieces of the liver specimens were excised carefully from both the control and experimentally treated rats, fixed in 10% neutral formalin, and dehydrated by repeated exposure to serial alcohol concentrations. The samples were cleared using xylene and infiltrated and embedded in paraffin wax, forming blocks according to the routine processing protocol of Bancroft and Gamble [126]. Using a rotary microtome, sections 5 μm in thickness were cut. Hematoxylin and eosin (H&E) staining was used to examine the tissue sections using Olympus CX22 light microscopy to detect histopathological changes.

### 4.8. Transmission Electron Microscopic Investigation

Small pieces of liver tissues of the control and treated rats were immediately fixed in 4% formalin and 1% glutaraldehyde (4F1G, fixative mixture) and rinsed in phosphate buffer (0.1 M, pH 7.4) at 4 °C for 24 h. Then, post-fixation using 1% buffered Osmium tetroxide (OsO4) at 4 °C for 2 h was performed. The samples were washed for 30 min in phosphate buffer and dehydrated through ascending grades of ethanol concentrations at 4 °C. The specimens were treated with propylene oxide and embedded in an Epon–Araldite resin mixture (1:1) [127]. Ultrathin sections (60 nm thick) were obtained by cutting with a glass knife on LKB ultra-microtome, mounted on 200 mesh naked copper grids, and double-stained with uranyl acetate and lead citrate for 30 min. Finally, the samples were examined and photographed using a JEM-1400 Plus Transmission Electron Microscope (TEM) (Faculty of Science, Alexandria University, Alexandria, Egypt).

### 4.9. Statistical Analysis

Data were given as a mean ± SD. One-way ANOVA was used to analyze the statistical differences within groups, followed by a post hoc test (Tukey) to detect the significance between each two groups. The significance of the results was judged at the 5% level (*p* ≤ 0.05). These statistical analyses were performed using version 24.0 of IBM’s SPSS software (Chicago, IL, USA).

## 5. Conclusions

Exposure to UVC radiation exerts toxic effects on rat livers. Pretreatment with vitamins C and B12 is essential to overcome the hazardous effect of UVC radiation. Vitamins restore most normal hepatic structures by improving the antioxidant levels and decreasing oxidative stress due to their free radical scavenger effect, compared to UVC-irradiated rats. Vitamin C was more effective in modulating the inflammatory markers and hepatic injury of UVC than vitamin B12. Vitamins C and B12 can be used as prophylactic tools for workers in UVC disinfectant areas. The estimated mean daily intake of vitamins C and B12 were 70–120 mg and 4–6.2 µg, respectively, for adults. These quantities are easily affordable with a well-balanced diet. The recommended daily intake of vitamins varies depending on the life stage and even the country.

## Figures and Tables

**Table 1:** Primer sequences, RT-PCR conditions, and product sizes.

Gene Name/Size/ Accession Number	Forward	Reverse	Annealing Tm (°C)
**GAPDH/309/** **NM_017008.4**	TCCCTCAAGATTGTCAGCAA	AGATCCACAACGGATACATT	**52**
**TNF-α/235/** **NM_012675.3**	ACACACGAGACGCTGAAGTA	GGAACAGTCTGGGAAGCTCT	**52**
**IL-1β/104/** **NM_031512.2**	GACTTCACCATGGAACCCGT	GGAGACTGCCCATTCTCGAC	**52**
**IDO-1/256/** **NM_002164.6**	TGGCAAGACCTTACGGACATCTC	AGAAGTGGGCTTTGCTCTGC	**60**
**iNOS/314** **NM_012611.3**	GGACCACCTCTATCAGGAA	CCTCATGATAACGTTTCTGGC	**60**

GAPDH: Glyceraldehyde-3-phosphate dehydrogenase; TNF-α: Tumor necrosis factor-α; IL-1β: Interleukin-1β; IDO-1: Indoleamine 2,3 dioxygenase-1; iNOD: Inducible nitric oxide synthase.

## Data Availability

The datasets generated during the current study are available from the corresponding author upon reasonable request.

## Supplementary Materials

The following supporting information can be downloaded at: https://www.mdpi.com/article/10.3390/molecules28114302/s1.

## Sample Availability

The compounds used in the study are not available from the authors.
