# Supplementary material for: The Prophylactic Effect of Vitamin C and Vitamin B12 against Ultraviolet-C-Induced Hepatotoxicity in Male Rats
Source: Molecules. 2023 May 24;28(11):4302. doi: 10.3390/molecules28114302 (PMC10254172; doi:10.3390/molecules28114302)
Supplement: Supplementary file 1 [file molecules-28-04302-s001.zip › molecules-2336543-supplementary.pdf]

UVC lamp calibration:

UVC lamps have been calibrated in the National Institute of Standards – NIS Radiometry Lab. Reference Radiometry S480/268-UVC – Report No. 79/52/2022, Cairo. The experimental sets up are the timer and the power supply controller. The timer managed the UV exposure time (8 hours/day). The power supply controller checks and controls the power supply's steady stability during the exposure period. It gives a measure for the whole exposure time over the experiment period. One-hour feeding break was provided at mid-day.

The average exposure doses to UVC radiation with daily interval exposure (8 hours/day) for 14 days were measured to be 725.76, 967.68, and 1048.32 J/cm^2. Representing low, mild, and high doses, respectively.

The UV light lamps were measured to have a wavelength of 254 nm.

Time of one-day exposure = 8hr. x 60 min. x 60 sec. =28800 sec.
Time of 14- days exposure = 403200 sec.
Adjusted distance = 30 cm

The Calculations:

Irradiance of one lamp source = (0.91+0.59+0.30) =1.800 $mW/cm^2/sec$.

Total Dose for 14 days exposure = 1.800 x403200 = 725760 mJ/cm^2

=725.760 J/cm^2

Irradiance of two lamps = (1.2+0.8+0.4) =2.4 $mW/cm^2/sec$.

Total Dose for 14 days exposure = 2.4 x403200 = 967680 mJ/cm^2

=967.680 J/cm^2

Irradiance of three lamps = (1.3+0.9+0.4) =2.6 $mW/cm^2/sec$.

Total Dose for 14 days exposure = 2.6 x403200 = 1048320 mJ/cm^2

=104.8320 J/cm^2

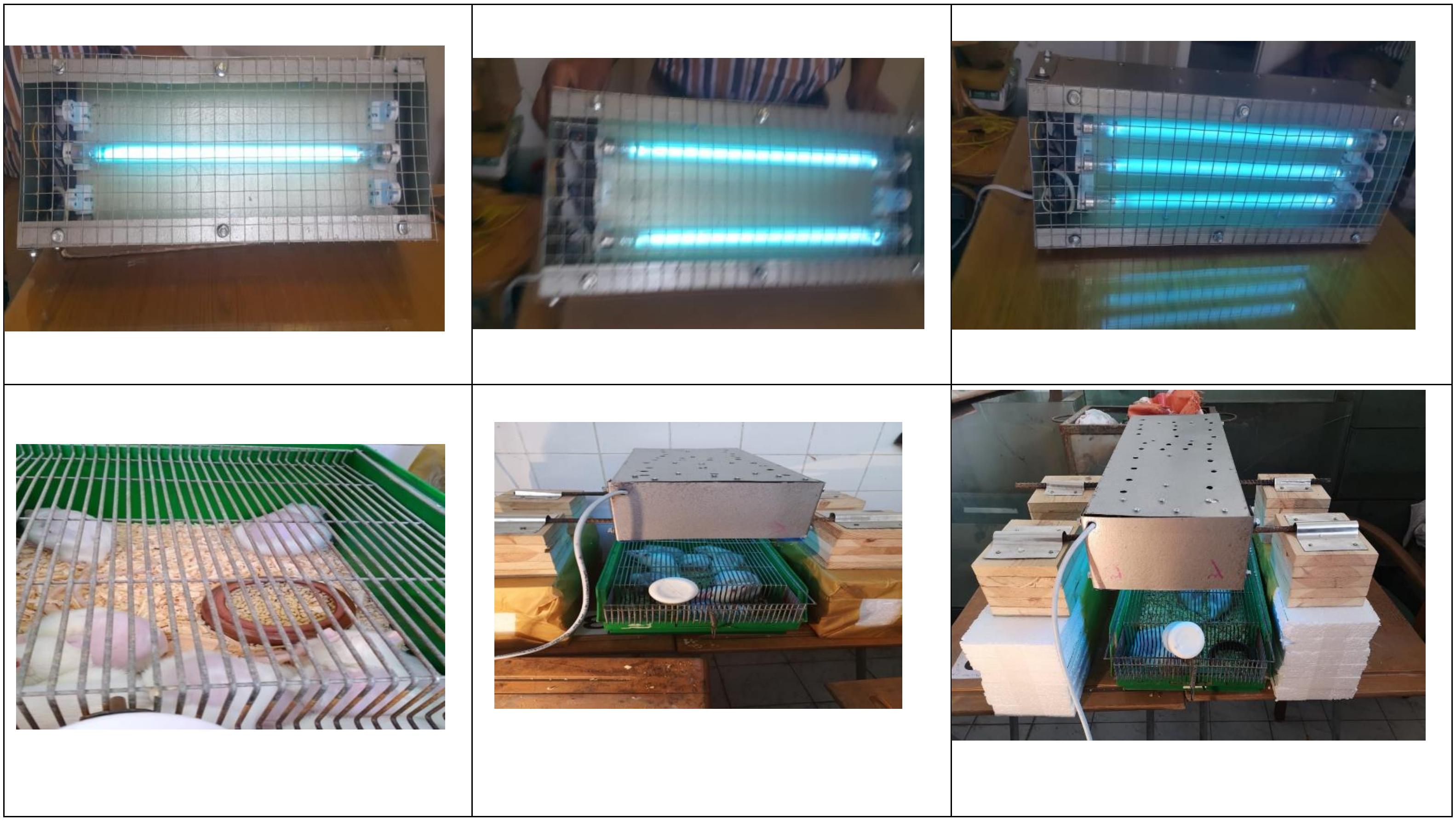

Figure S1. The experimental setup
